# A five-country comparison of midwifery students' confidence in facilitating normal labor and birth

**DOI:** 10.18332/ejm/210325

**Published:** 2025-10-17

**Authors:** Juliet Wood, Jalana Lazar, Barbara Baranowska, Clare Davison, Debora Dole, Cindy Farley, Jane Fry, Maria Healy, Felicity Agwu Kalu, Urszula Tataj-Puzyna, Emma Ritchie, Maria Wegrzynowska

**Affiliations:** 1 Department of Midwifery and Health Sciences, Bournemouth University, Bournemouth, United Kingdom; 2 Nurse-Midwifery/Women’s Health Nurse Practitioner Program, Berkley School of Nursing, Georgetown University, Washington, United States; 3 Department of Midwifery, Centre of Postgraduate Medical Education, Warsaw, Poland; 4 Edith Cowan University, Joondalup, Australia; 5 School of Nursing and Midwifery, Queen's University Belfast, Belfast, Northern Ireland; 6 College of Nursing and Midwifery, Charles Darwin University, Darwin, Australia

**Keywords:** midwifery education, student confidence, normal birth, physiologic birth, midwifery care

## Abstract

**INTRODUCTION:**

Midwifery students need confidence in recognizing and supporting normal birth, the backbone of the midwifery professional role. Developing this confidence in the face of decreasing rates of physiological birth worldwide is a critical challenge. Midwife researchers from Australia, England, Northern Ireland, Poland, and the USA investigated midwifery student confidence for supporting normal birth and explored enhancing and detracting factors.

**METHODS:**

A cross-sectional survey design was undertaken with 570 midwifery students at 8 academic midwifery programs across 5 countries The Student Confidence for Supporting Normal Birth Questionnaire with free text and Likert-type questions on a 1 (least influential) to 4 (most influential) scale was used. The survey was distributed between 2019 and 2023. Quantitative data were analyzed using descriptive statistics and Kruskal-Wallis tests of difference. Free text responses were analyzed thematically.

**RESULTS:**

Overall confidence mean was 2.06/4.00, with Poland (1.67) having the lowest confidence and the USA the highest (2.88). Factors rated most influential were the student–mentor midwife relationship (3.40) and theoretical education (3.09). In addition, birth environment emerged as important in the qualitative themes.

**CONCLUSIONS:**

Interacting with a mentor midwife that supports physiological birth and is respectful of students, and repeated exposure to birth environments that privilege women-centered physiological birth are crucial to ensuring midwifery students can transition to confident midwifery professionals who are advocates for physiological birth. Didactic education that emphasizes the basic physiological and psychological principles that underlie midwifery care processes, contributes to midwifery student confidence for supporting normal birth.

## INTRODUCTION

Global policy recommendations support growing and strengthening the midwifery workforce as the midwifery model of care has been shown to improve maternal–child health outcomes^1,2^. Simultaneously, international efforts are underway to support countries in achieving worldwide standards of quality midwifery care. The International Confederation of Midwives (ICM) defines the midwifery philosophy as viewing ‘pregnancy, birth and postpartum as normal and profound life experiences, and stresses the role of the midwife in supporting normalcy. Midwives optimize physiological processes and support safe physical, psychological, social, cultural and spiritual situations, working to promote positive outcomes and to anticipate and prevent complications’^3^. Indeed, guardianship of physiological birth is central to the midwifery model of care and quality maternal and newborn care.

Midwives cite supporting normal, physiological birth as a core professional tenet, and midwifery educators understand it to be fundamental to the professional development of midwifery students^4^. Limited research suggests that student confidence in supporting physiological birth is important to midwifery practice and can be modified^5-7^. However, the clinical environments in which many midwifery students are being educated reflect national declines in physiological labor and birth and rising rates of routine elective medical intervention^8-15^. Furthermore, in many countries, midwives are constrained by regulatory environments and healthcare cultures that do not fully support autonomous midwifery practice and thus limit implementation of midwifery models of care. Effectively enabling students to develop skills and confidence to support physiological labor and birth is challenging.

In 2019, midwife educators from five countries (Australia, England, Northern Ireland, Poland, and the USA) developed a collaborative study investigating midwifery students’ confidence in supporting physiological birth. All the researchers in this study are teaching curricula designed to prepare midwives to meet national and international standards of care that endorse support of physiological labor and birth. Whilst programs of midwifery education vary within and between each of the five participating countries, all registered midwives meet the ICM criteria for educational preparation and core competencies of a midwife. Midwives in Australia, England, and Northern Ireland are among lead maternity professionals for healthy childbearing women with straightforward pregnancies^16,17^. In the USA and Poland, midwifery is often subordinated to obstetrics, with midwives attending only 12% of births in the US or being largely excluded from providing antenatal care in Poland^18,19^. Yet in each country, standards of midwifery competence and care and midwifery curricula reflect the value of normal, physiological birth.

The two concepts of interest in this study are normal, physiological birth and student confidence. The ICM defines physiological birth as a dynamic life process that occurs when a woman or gender diverse person starts, continues, and completes labor and birth spontaneously at term with the fetus in vertex position and experiences no surgical, medical, or pharmaceutical intervention^3^. Confidence is often conceptualized as self-efficacy, a theoretical precept of Bandura’s Social Learning Theory^20^. Self-efficacy is a dynamic cognitive process that is an individual’s belief in her/his abilities to perform required behaviors in novel or stressful situations. Sources of self-efficacy include performance accomplishment, vicarious experience, verbal persuasion, and visceral arousal. Self-efficacy is a major determinant of the degree of effort and persistence that will be applied to the task at hand. Intervening variables affecting student confidence for supporting normal labor and birth can include disincentives to act upon one’s selfefficacy beliefs and new experiences that can prompt reappraisals of one’s abilities in the time prior to action^21^.

This study explored levels of student confidence and identified barriers and facilitators to midwifery students’ confidence in supporting normal birth. Findings were compared and contrasted across five countries.

## METHODS

### Design and settings

A cross-sectional exploratory survey research design was used to investigate student midwife perceptions regarding factors that influence their confidence to support normal labor and birth.

### Survey

A researcher-designed survey was developed from literature published on student confidence to support normal birth and from social learning theoretical precepts. It included 3 open-ended questions and 11 Likert-type questions, scored from 1 (least influential) to 4 (most influential). Likert scale questions were followed by a free text invitation to add information regarding the numeric response. The directionality and emphasis of the influence of the variables queried were given context through the free text responses available with each Likert scale response. The questionnaire was pilot tested on a small number of students to check for content validity and acceptability, and minor edits were undertaken. Face and content validity was ascertained by expert review and pilot participant input. Some word choices on the questionnaire were adapted to fit local language and maternity service provision in each of the participating countries. For example, the word ‘preceptor’ is typically used in the USA to indicate the clinical educator role, ‘supervisor’ is used in England and Northern Ireland, and ‘mentor’ is used in other participating countries. The Polish questionnaire was translated bidirectionally to assure clarity and comparability of the survey across the Englishspeaking countries.

The literature was searched for factors associated with student midwife confidence, supporting the questions that were developed (Table 1). The Likert scale questions were scaled from 1 to 4, with 1 being least influential and 4 being most influential to confidence. In the open-ended questions, respondents were asked to list the three most important factors which increased their confidence to maintain or promote normal, physiological labor and birth and the three which most decreased it. The final free text question asked respondents to explain what the phrase ‘normal labor and birth’ meant to them.

**Table 1:** Factors from literature and expert review influencing student midwife confidence for supporting normal labor and birth included in the questionnaire

Woman’s/birthing person’s attitude towards birth
Place of birth
Physical environment of birth
Role of theoretical education about physiological labor and birth
Influence of the mentor/supervisor/preceptor midwife overseeing the student’s practice
Influence of national/state labor and birth policies
Influence of local/hospital labor and birth policies
Staffing levels
Possibility of providing continuity of carer
Influence of other staff (e.g. doctors, managers) or midwives other than the mentor/supervisor/preceptor)

### Ethics

Ethical approval was obtained from the Bournemouth University Research Ethics Committee (REC reference: 24299) and thereafter each country research team applied to their own local research ethics group and received approval. Participants were guaranteed anonymity and the freedom to withdraw at any point. Consent was indicated through completion of the online survey. Student responses were anonymized; thus, potential risks of student vulnerability were mitigated.

### Participants

A convenience sampling strategy was employed. The survey was introduced to cohorts of students during class or by e-mail, and the deadline for close of the survey was highlighted. The QR code or link to the survey was emailed to each eligible student and two reminder emails were sent to maximize participation. The timing of the survey administration was staggered as midwife educators from the various countries joined the research team at different times, thus data collection occurred between 2019 and 2023. The anonymized survey was disseminated and completed by the student respondents across cohorts in each country. Response rates ranged from a high in Poland (73.4%; n=219/298) to a low in Australia of 21%; n=37/174) (Supplementary file Table 1). Midwifery students from 8 universities in 5 countries participated. Participating midwifery education program characteristics are found in Supplementary file Table 2.

### Data analysis

Data from all countries were cleaned, entered, and analyzed using IBM SPSS Statistics (Version 29.0.1.0). Frequencies and percentages, and means and standard deviations, were determined. Additionally, respondents were categorized into early, mid or late phases of education and compared. These categories were sorted by the country teams based on their curricula. The US is unique in that their early phase, students are pre-clinical. Clinical experiences are not introduced in the US until the mid-phase of their curriculum, whereas the other participating countries all introduce selected clinical experiences early in their programs. Tests of statistical difference were applied across the different cohorts within each country and across the countries. As the data were not normally distributed, the Kruskal-Wallis test was used for testing the differences in means. This test was applied in two ways for each variable: differences between the country means were tested and for each country, differences in the means between the phases of the midwifery student cohorts were also tested.

For the free text questions on the top three enablers and inhibitors for developing confidence, the responses were grouped independently by members of the research team using reflexive thematic analysis^22^. Emerging themes were compared, consensus was achieved, and the relative importance of each enabler or inhibitor within each country was assigned a value to allow comparison across countries.

## RESULTS

A total of 570 students responded. Table 2 is a summary of the survey data of each country that participated in the study. Respondents from all countries were in various years of their midwifery studies (Supplementary file Table 2). Midwifery students were categorized according to the country where they attended their midwifery education. Countries, such as England and Northern Ireland, often accept students from other countries, such as Spain, Italy, Nigeria, and the United Arab Emirates, while international students are uncommon in midwifery education programs in Australia, Poland, and the USA. Students in all countries were predominantly female and White. During the study period, the age range of midwifery students was from 18 years up to the 50s. The US was the exception, with ages ranging 25–40 years. The most common educational pathway to midwifery in the US requires the applicant to be a Registered Nurse with a Bachelor’s degree, while the other participating countries accept eligible students with appropriate academic qualifications after post-secondary education.

**Table 2:** Mean differences in midwife student confidence for supporting normal birth by country

*Items*	*Poland* *Mean* *SD*	*England* *Mean* *SD*	*USA* *Mean* *SD*	*Northern* *Ireland* *Mean* *SD*	*Australia* *Mean* *SD*	*Five* *countries* *Mean* *SD*	*Normality* *of residuals* *W-stat* *p ^g^*	*Homogeneity* *of variances* *F* *p ^h^*	*Differences* *of means* *K* *p ^i^*
**Total,** n	219	162	85^a^	67^b^	37	570^c^			
**How confident do you feel to maintain or promote normality in labor and birth?^d^**	1.67	2.08	2.88	2.24	2.16	2.06	0.852*	12.654*	100.297*
	0.756	0.722	1.011	0.836	0.800	0.896	<0.000	<0.000	<0.0001
**How much influence have mentors (or a particular mentor) (US=preceptors) in practice had on your confidence and ability to promote or maintain normal birth?^f^**	3.42	3.41	3.33	3.49	3.14	3.40	0.744*	0.209	5.673
	0.727	0.744	0.818	0.683	0.855	0.748	<0.000	0.934	0.225
**Rate how much influence staffing levels have on your confidence and ability to promote or maintain normal birth.^f^**	3.60	2.95	3.04	3.55	3.22	3.31	0.766*	38.951*	108.659*
	0.568	0.924	0.794	0.661	0.886	0.796	<0.000	<0.000	<0.0001
**How much influence do other personnel (for example, obstetricians, midwifery managers, other midwifery colleagues and maternity support workers) have on your confidence and ability to promote or maintain normal birth?^f^**	3.50	3.13	2.99	3.33	3.41	3.27	0.792*	0.407	53.647*
	0.687	0.773	0.703	0.771	0.686	0.772	<0.000)	0.840	<0.0001
**How much influence has your theoretical learning had on your confidence and ability to promote or maintain normal birth?^f^**	3.35	2.95	2.98	2.91	2.76	3.09	0.831*	0.372	38.120*
	0.735	0.847	0.913	0.866	0.723	0.836	<0.000	0.829	<0.0001
**Rate how much influence continuity of care has on your confidence and ability to promote or maintain normal birth.^f^**	3.16	3.14	2.85	2.55	3.51	3.09	0.829*	5.515*	29.554*
	0.820	0.803	0.794	1.061	0.692	0.860	<0.000	0.0002	<0.0001
**How much influence do Local Labor and Birth Policies (US=hospital labor and birth policies) have on your confidence and ability to promote or maintain normal birth?^f^**	3.09	2.84	3.12	3.03	2.86	2.91	0.843*	1.871	9.607*
	0.805	0.898	0.762	0.887	0.918	0.915	<0.000	0.114	0.048
**To what extent does the woman’s attitude towards birth affect your confidence an ability to maintain or promote normality in labor and birth?^e^**	2.98	2.73	2.69	2.79	3.11	2.85	0.858*	2.226	16.123*
	0.815	0.810	0.776	0.930	0.843	0.832	<0.000	0.065	0.003
**To what extent does the physical location of birth impact your ability to promote or maintain normal birth?^e^**	2.60	2.77	2.65	2.69	3.03	2.69	0.868*	5.899*	6.67
	1.028	0.900	0.869	1.037	0.763	0.959	<0.000	0.0001	0.155
**To what extent does the physical environment of birth (e.g. the layout of the birthing room, the equipment available) impact your ability to promote or maintain normal birth?^e^**	2.81	2.53	2.27	2.58	2.68	2.62	0.877*	3.755*	23.290*
	0.816	0.920	0.892	0.987	0.747	0.892	<0.000	0.005	<0.000
**How much influence do National Labor and Birth Policies (US=State labor and birth laws and policies) have on your confidence and ability to promote or maintain normal birth?^f^**	2.61	2.58	2.42	2.97	2.41	2.60	0.879*	4.058*	150.311*
	0.909	0.876	1.084	0.870	0.985	0.938	<0.000	0.003	0.007

a For the US sample, the variable on confidence n=84, those on the influence of women n=84, mentors/preceptors n=55.

b For the NI sample, the variable on the influence of birth location n=64, those on staffing levels n=66, on continuity of care n=65, on other colleagues n=66.

c Influencing factor variables arranged in decreasing order of the means from the full five-country sample.

d 1=not at all, 2=occasionally, 3=often, 4=always.

e 1=no influence, 2=occasional influence, 3=it often influences, 4=it always influences.

f 1=no influence, 2=occasional influence, 3=it often has an influence, 4=it always has an influence.

g Shapiro-Wilk test.

h Levene test.

i Kruskal-Wallis test.

* Significant at the 5% level.

### Confidence to maintain or promote normality in labor and birth

The mean response for the main question ‘How confident do you feel to maintain or promote normality in labor and birth?’ for the five-country sample was 2.06 (p=0.896), just above the score for the second lowest response option of ‘fairly confident’ (Table 2). The country means varied from 1.67 for Poland to 2.88 for the USA. The mean scores for Northern Ireland, Australia and England are within a narrow range in the middle (2.24, 2.16, and 2.08 respectively).

The mean scores for confidence by country and by phase of education are shown in Table 3. For five countries, the mean for the late cohort is the highest, while the mean for the early cohort is the lowest or equal lowest, except in the USA. The mid cohort in the USA has the lowest mean across its phases, although the late cohort shows a rebound in confidence with a mean higher than the early cohort. Only in England are the differences in means between the early, mid and late cohorts statistically significant, showing an upward trend in mean confidence. The differences in means for the combined sample have p=0.051, just above the 5% level.

**Table 3:** Comparison of means in midwife student confidence for supporting normal birth by phase of midwifery education and by country^a^

*Items*	*Poland (N=219)*				*England (N=162)*				*USA (N=85)*				*Northern Ireland (N=67)*				*Australia (N=37)*				*Five countries (N=570)*			
	*Early* *M* *SD* *R*	*Mid* *M* *SD* *R*	*Late* *M* *SD* *R*	*Diff. of* *means* *K* *p ^f^*	*Early* *M* *SD* *R*	*Mid* *M* *SD* *R*	*Late* *M* *SD* *R*	*Diff. of* *means* *K* *p ^f^*	*Early* *M* *SD* *R*	*Mid* *M* *SD* *R*	*Late* *M* *SD* *R*	*Diff. of* *means* *K* *p ^f^*	*Early* *M* *SD* *R*	*Mid* *M* *SD* *R*	*Late* *M* *SD* *R*	*Diff. of* *means* *K* *p ^f^*	*Early* *M* *SD* *R*	*Mid* *M* *SD* *R*	*Late* *M* *SD* *R*	*Diff. of* *means* *K* *p ^f^*	*Early* *M* *SD* *R*	*Mid* *M* *SD* *R*	*Late* *M* *SD* *R*	*Diff. of* *means* *K* *p ^f^*
**Total,** n	31	116	72		66	59	37		39^b^	33^b^	13^b^		13	33	21		15	15	7					
**How confident do you feel to maintain or promote normality in labour and birth?^c^**	1.55	1.6	1.82	5.465	1.91	2.14	2.30	7.680*	2.89	2.67	3.38	5.409	2.31	1.97	2.62	7.078	2.00	2.00	2.33	1.898	2.11	1.93	2.22	10.268*
	0.72	0.76	0.76	0.065	0.91	0.75	0.76	0.021	1.16	0.85	0.77	0.067	0.85	0.77	0.80	0.029	1.00	0.53	0.77	0.387	0.99	0.82	0.88	0.006
	1–3	1–4	1–4		1–4	1–4	1–4		1–4	1–4	2–4		1–4	1–4	1–4		1–4	1–3	1–4		1–4	1–4	1–4	
**How much influence have mentors or a particular mentor (US=preceptors) in practice had on your confidence and ability to promote or maintain normal birth?^e^**	3.48	3.44	3.36	2.084	3.32	3.47	3.46	0.593	3.08	3.33	3.58	1.246	3.46	3.48	3.52	0.263	2.55	3.38	3.39	8.102*	2.85	2.80	2.93	1.649
	0.81	0.74	0.68	0.353	0.86	0.60	0.66	0.743	1.12	0.76	0.51	0.536	0.66	0.71	0.68	0.877	0.82	0.74	0.78	0.017	0.85	0.84	0.81	0.438
	1–4	1–4	1–4		1–4	2–4	2–4		1–4	1–4	3–4		2–4	2–4	2–4		1–4	2–4	1–4		1–4	1–4	1–4	
**Rate how much influence staffing levels have on your confidence and ability to promote or maintain normal birth.^e^**	3.68	3.59	3.60	2.330	3.08	2.86	2.86	3.383	3.31	2.82	2.77	8.380*	3.38	3.73	3.35	6.177	3.18	3.25	3.22	0.240	2.71	2.64	2.78	3.387
	0.7	0.53	0.57	0.312	1.00	0.82	0.90	0.184	0.69	0.85	0.73	0.015	0.51	0.57	0.81	0.046	0.75	1.04	0.94	0.887	0.92	0.98	0.95	0.184
	1–4	2–4	1–4		1–4	1–4	1–4		2–4	1–4	2–4		3–4	2–4	2–4		2–4	1–4	1–4		1–4	1–4	1–4	
**How much influence do other personnel (e.g. obstetricians, midwifery managers, other midwifery colleagues and maternity support workers) have on your confidence and ability to promote or maintain normal birth?^e^**	3.61	3.47	3.50	1.705	3.20	3.08	3.08	0.845	2.95	3.03	3.00	0.147	3.38	3.52	3.00	4.469	3.18	3.75	3.39	3.156	2.51	2.69	2.60	0.411
	0.67	0.67	0.73	0.426	0.75	0.77	0.82	0.655	0.84	0.59	0.58	0.929	0.77	0.62	0.92	0.107	0.75	0.46	0.70	0.206	0.91	0.86	0.91	0.814
	1–4	2–4	1–4		1–4	2–4	1–4		1–4	2–4	2–4		2–4	2–4	1–4		2–4	3–4	2–4		1–4	1–4	1–4	
**How much influence has your theoretical learning had on your confidence and ability to promote or maintain normal birth?^e^**	3.42	3.35	3.32	0.949	2.94	2.90	3.05	0.989	2.79	3.03	3.38	3.765	2.92	2.94	2.86	0.393	2.55	3.00	2.78	2.515	2.97	3.14	3.14	2.821
	0.85	0.68	0.78	0.622	0.89	0.78	0.86	0.610	1.03	0.77	0.77	0.152	0.64	0.93	0.91	0.822	0.82	0.53	0.73	0.284	0.92	0.77	0.84	0.244
	1–4	2–4	1–4		1–4	1–4	1–4		1–4	1–4	2–4		2–4	1–4	1–4		2–4	2–4	2–4		1–4	1–4	1–4	
**Rate how much influence continuity of care has on your confidence and ability to promote or maintain normal birth.^e^**	3.45	3.2	2.96	9.619*	3.15	3.15	3.11	0.216	2.97	2.76	2.69	2.810	2.62	2.56	2.50	0.037	3.73	3.75	3.28	3.193	3.28	3.44	3.43	9.991*
	0.81	0.78	0.85	0.008	0.85	0.76	0.80	0.898	0.84	0.71	0.86	0.245	0.96	1.08	1.15	0.982	0.47	0.46	0.83	0.203	0.88	0.70	0.69	0.007
	1–4	1–4	1–4		1–4	1–4	2–4		1–4	2–4	1–4		1–4	1–4	1–4		3–4	3–4	2–4		1–4	1–4	1–4	
**How much influence do Local Labour and Birth Policies (US=hospital labour and birth policies) have on your confidence and ability to promote or maintain normal birth?^e^**	3.13	3.06	3.11	0.464	2.80	2.92	2.78	0.790	2.95	3.3	3.15	3.048	3.31	2.91	3.05	1.808	2.91	2.75	2.89	0.140	2.61	2.60	2.60	2.402
	0.88	0.81	0.78	0.793	0.85	0.92	0.97	0.674	0.86	0.64	0.69	218	0.75	0.88	0.97	0.405	1.04	1.04	0.83	0.932	0.96	0.92	0.94	0.301
	1–4	1–4	1–4		1–4	1–4	1–4		1–4	2–4	2–4		2–4	1–4	1–4		1–4	1–4	1–4		1–4	1–4	1–4	
**To what extent does the woman’s attitude towards birth affect your confidence and ability to maintain or promote normality in labour and birth?^d^**	3.16	2.87	3.07	4.378	2.77	2.59	2.89	3.961	2.68	2.73	2.62	0.070	2.54	3.00	2.62	3.455	3.36	2.88	3.06	1.301	2.85	2.95	2.93	2.383
	0.78	0.81	0.83	0.112	0.87	0.79	0.74	0.138	0.81	0.72	0.87	0.966	0.88	1.00	0.80	0.178	0.67	1.13	0.80	0.522	0.95	0.89	0.92	0.304
	2–4	1–4	1–4		1–4	1–4	2–4		1–4	1–4	1–4		1–4	1–4	1–4		2–4	1–4	2–4		1–4	1–4	1–4	
**To what extent does the physical location of birth impact your ability to promote or maintain normal birth?^d^**	2.81	2.51	2.67	1.996	2.74	2.71	2.92	1.407	2.62	2.82	2.31	3.031	1.77	2.80	3.10	3.766	3.36	2.75	2.94	3.766	3.19	3.39	3.32	2.122
	0.87	1.1	0.96	0.369	0.92	0.89	0.94	0.495	0.88	0.81	0.95	0.220	0.93	0.89	1.00	0.178	0.67	0.46	0.87	0.152	0.89	0.72	0.80	0.346
	1–4	1–4	1–4		1–4	1–4	1–4		1–4	1–4	1–4		1–4	1–4	1–4		2–4	2–3	1–4		1–4	1–4	1–4	
**To what extent does the physical environment of birth (i.e. the layout of the birthing room, the equipment available) impact your ability to promote or maintain normal birth?^d^**	2.87	2.84	2.75	0.623	2.45	2.54	2.65	0.791	2.31	2.33	2.00	1.300	2.31	2.76	2.48	1.828	2.82	2.88	2.50	1.852	3.25	3.07	2.96	1.011
	0.81	0.82	0.82	0.732	0.95	0.86	0.91	0.673	0.92	0.89	0.82	0.522	0.95	0.94	1.08	0.401	0.60	0.64	0.86	0.396	0.82	0.85	0.89	0.603
	1–4	1–4	1–4		1–4	1–4	1–4		1–4	1–4	1–3		1–4	1–4	1–4		2–4	2–4	1–4		1–4	1–4	1–4	
**How much influence do National Labor and Birth Policies (US=State labor and birth laws and policies) have on your confidence and ability to promote or maintain normal birth?^e^**	2.58	2.56	2.72	1.807	2.65	2.58	2.46	1.345	2.59	2.42	1.92	3.696	3.08	2.91	3.00	0.479	2.00	2.88	2.44	3.637	3.24	3.30	3.26	0.023
	1.03	0.89	0.89	0.405	0.83	0.88	0.95	0.511	1.07	1.09	1.04	0.158	0.95	0.88	0.84	0.787	1.00	1.13	0.86	0.162	0.78	0.74	0.82	0.989
	1–4	1–4	1–4		1–4	1–4	1–4		1–4	1–4	1–4		1–4	1–4	2–4		1–4	1–4	1–4		1–4	2–4	1–4	

M: mean. SD: standard deviation. R: range. Diff.: differences.

a Influencing factor variables arranged in decreasing order of the means from the full five-country sample.

b For the US sample, the variable on confidence n=84, those on the influence of women n=84, mentors/preceptors n=55.

c 1=not at all, 2=occasionally, 3=often, 4=always.

d 1=no influence, 2=occasional influence, 3=it often influences, 4=it always influences.

e 1=no influence, 2=occasional influence, 3=it often has an influence, 4=it always has an influence.

f Kruskal-Wallis test.

* Significant at the 5% level.

In summary, midwifery students’ confidence in their ability to maintain and promote physiological labor and birth, tends to increase within each country as their education advances. The USA is the only exception to this pattern. There are high confidence levels among pre-clinical respondents in the US sample. These levels contribute somewhat to the USA having the highest overall mean among the five countries, but the USA mean is 2.73, still well above those of Northern Ireland (2.24), Australia (2.16) and England (2.08). The relatively low score of Poland (1.67) is another outlier.

### Factors which influence the confidence to maintain or promote physiological labor and birth

Table 2 shows the country means for the ten Likert scale questions regarding factors which may influence midwifery students’ confidence to maintain or promote normality in labor and birth. The column that shows the 5-country means is arranged in order of decreasing means. Within each country sample, the top four factors are shaded in decreasing order of intensity corresponding with decreasing means.

Among the top four factors influencing student confidence in supporting normal birth overall, the impact of clinical midwifery staff predominates. The top three factors are mentors (preceptors/supervisors), overall staffing levels, and other personnel, while the fourth relates to theoretical education.

There are notable similarities across countries in the most important influencing factors. Mentors, personnel and staffing were top-ranking influential factors in every country. However, there were unique differences as well. Continuity of care was rated higher in England and Australia compared to the other countries. Local labor and birth policies, which were not in the top four ranking factors in the overall analysis, were among the top four ranking choices for Northern Ireland and the USA. The mean of the top-ranking impact measure, mentors, was not significantly different between countries (p=0.748).

For all other influencing factors, there was less consistency; the mean ranks are significantly different. However, the range of mean scores for the influencing factors across countries tended to be smaller than was the case for confidence levels, though ‘continuity of care’ was an exception with two outliers. Comparison of the country-level rankings of influencing factors offers an alternative comparison which is less prone to confounding influences of unobserved contextual differences between countries. Each country displays a unique ranking of the ten influencing factors.

Table 3 shows the mean values for the 10 influencing factors by phase of education and by country. As in Table 2, they are arranged in the same order, namely by decreasing mean, for the combined 5 country analysis. Differences in the mean ranks by phase of education were found to be significant for only three influencing factors and for a single country in each case. These factors were mentors, staffing levels (the top two ranked factors) and continuity of care.

### Qualitative analysis of free text responses

Free text responses collected from the two open-ended questions concerning aspects that facilitated or hindered students’ confidence in supporting physiological birth aligned with the quantitative findings described above, this is notable given the open-ended questions were asked before the Likert questions suggesting impacting factors. Analysis of these data generated three main content themes: birth environment and exposure to physiological birth; the relationship between a student midwife and a mentor/supervisor/preceptor midwife; and theoretical education.

**Figure 1:**
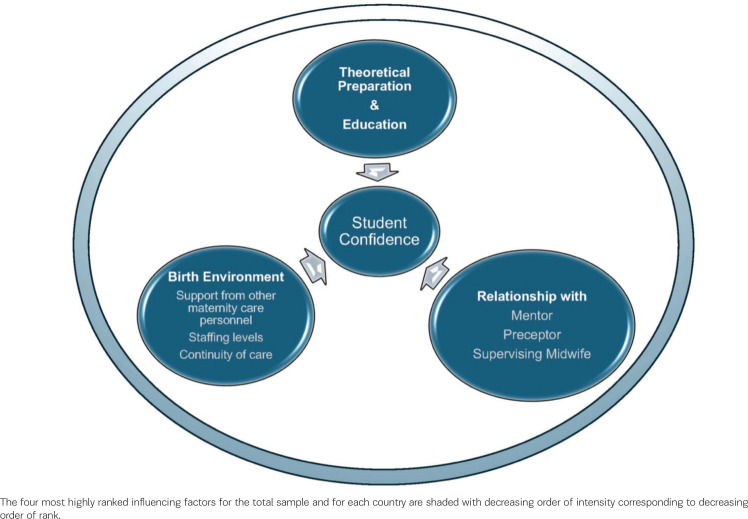
Integrated factors that influence student midwife confidence in supporting physiological birth

What students considered as normal birth varied not only across countries, but also among students from each country in the responses to that open-ended question. Whilst most students reported that normal birth is a vaginal birth, with no or minimal interventions, spontaneous in onset, involving respectful care, supported by midwife and resulting in a healthy mother and baby, the concept of normal birth was disputed. Some students reported that all births are normal; for others, it was the degree of intervention used, with the use of minimal intervention still considered normal. However, what constituted ‘minimal interventions’ to students was not made clear in their responses.

### Birth environment and exposure to normal birth

Students in their responses frequently referred to exposure to and personal experience with supporting normal birth as very important in building their confidence. This theme was particularly prominent in students’ answers from England, Australia, and Northern Ireland. For example, a third-year midwifery student from Australia shared:

*‘I worked for a month with the publicly funded home birth service. This open up my eyes to normal birth and that intervention is not normal unless it is required for the health and wellbeing of the mother and baby’.*

Working with midwives and childbearing individuals who trust the process of birth was also mentioned as supporting midwifery student confidence. When the midwife was indicated as a facilitator of student confidence, trust in the process and clinical skills were also mentioned as positive qualities of the mentor.

### The relationship between a student midwife and a mentor midwife

The quality of the relationship between midwifery students and a mentor (also known as preceptor or clinical supervisor or facilitator) midwife was found to be crucial in building students’ confidence to support normal birth. Having a mentor that believes in the woman’s ability to give birth was noted as confidence building to a secondyear midwifery student from England. This was reflected in both the quantitative findings, wherein the mentor midwife was ranked among the most important influences for students, and the qualitative findings. This theme of a positive relationship with the supervising midwife, or lack thereof, was particularly emphasized in the qualitative data from Poland (Table 4). Clinical mentors are not pre-assigned in Poland. When Polish students are reflecting on their mentors, they can be referring to a rotating roster of staff midwives working in a particular department where they are placed for their clinical learning.

**Table 4:** Free text responses from student midwives noting facilitators and barriers to their confidence for supporting normal birth

*Birth environment and exposure to physiological* *birth*	*The relationship between a* *student midwife and a mentor* *midwife*	*Theoretical education*
*‘Having the experience of normal births to fully understand intervention isn’t normal.’* (Third-year midwifery student, England) *‘Placement in midwifery-led unit provided an opportunity for me to learn and participate in the care of women having physiological birth.’* (Second-year midwifery student, Northern Ireland) *‘Being given time and space to care for the woman independently and minimal interruptions…enabled me and the midwife to support women giving birth.’* (Second-year midwifery student, Northern Ireland) *‘The fact that this is normal and it does not necessarily have to be medicalized. My clinical sites have a large volume of normal births, so I feel as though I am getting a ton of practice. I am becoming more and more able to recognize things as normal and abnormal.’* (Third-year midwifery student, USA) *‘Working on delivery suite. I had never seen one normal spontaneous labor. I am always allocated to woman undergoing inductions.’* (Second-year midwifery student, Australia)	*‘Having things explained, being encouraged to participate without fear of being criticized, told when things are done well.’* (First-year midwifery student, England) *‘Supportive and constructive feedback from midwives enabled me to learn how to care for women in labor.’* (Secondyear midwifery student, Northern Ireland) *‘Supportive team of midwives in the room made it comfortable and easier for me to ask questions.’* (First-year midwifery student, Northern Ireland) *‘It does not help when personnel just want to show me how little I know. They ask questions not to check my knowledge or educate me, but to humiliate me, they scream, they laugh at us and gossip about us when we are on the ward.’* (Second-year midwifery student, Poland)	*‘My theoretical learning is the main basis of my passion to promote and maintain normality as well as my own individual learning and practice experiences.’* (Third-year midwifery student, England) *‘Putting theory into practice during simulation practical classes within university has helped me.’* (Second-year midwifery student, Northern Ireland) *‘The education I have received from [my program], good clinical experience, and my own experience to promote normal in all aspects of healthcare as an RN.’* (Second-year midwifery student, USA) *‘Learning it in school, witnessing it in clinicals, learning different techniques in order to promote normal labor and birth.’* (Second-year midwifery student, USA)

Students mentioned mentor midwives’ empathy, willingness to share the knowledge, and receptivity to student questions as critical. Being practically involved in the care process and feeling needed in the team, was also frequently mentioned by students. However, a considerable number of students mentioned that they experienced bullying and mistreatment from the healthcare personnel and considered it to be a major barrier for them to enhance their confidence. They also mentioned negative criticism, lack of constructive feedback, and disregard of student questions from their midwife mentors as detracting from their confidence.

### Theoretical education

Students appreciated theoretical education and a strong background in the physiology of labor and birth, as it allowed them to better understand the process of birth and what they might encounter in practice:

*‘Thanks to theory, I know what consequences [of taking action] to expect and what I can do in certain situation.’* was expressed by a third-year midwifery student from Poland. However, some students mentioned experiencing a lack of coherence between the theoretical knowledge they received at the university and the practices they observed during their clinical placement. Our findings showed that students reported feeling more confident when they were fortified with theoretical and clinical midwifery education that supports physiological processes.

## DISCUSSION

This study examined midwifery student confidence in supporting normal birth and explored the influence of numerous elements on their confidence. Despite the different maternity health systems and regulatory bodies governing the practice of midwifery in each country and the varying midwifery curriculums delivered across five countries, there was a high degree of congruence across these diverse countries.

On average across countries, students rated themselves ‘fairly confident’ in their ability to support physiological birth, with students in the USA demonstrating slightly higher levels of self-confidence and Polish students slightly lower confidence levels. In four countries, student confidence increased as they progressed in their midwifery educational program; however, students in the US demonstrated a higher level of confidence measured prior to the start of their clinical rotations which then decreased after their first clinical experiences with labor and birth before rising again. It is interesting to note that Poland and the USA are the outliers in the confidence ranking. Poland (lowest confidence) has a medicalized system that constrains the autonomy of midwives and the USA (highest confidence) similarly has a medicalized system with numerous policy and facility constraints on midwifery autonomy. The midwifery students from the US were all Master’s degree students and already working as nurses before entering their training which may initially inflate their confidence ratings. However, when US midwifery students enter the clinical setting mid-program, they may find their confidence challenged by the realities of undertaking a midwifery role promoting physiological birth in a medicalized system of care.

In the case of Poland, it is postulated that low levels of confidence may be linked to the random and rotating assignments of mentors^23^. Most Polish midwifery education programs do not have clinical mentoring assignments that would allow individual midwifery students to have one or a few mentors working with them during each shift. While some universities have run pilot mentoring programs, most students work with different staff members during each clinical placement shift. If the midwife who is assigned to train a midwife student is overloaded with her/his own clinical responsibilities, they have little time to educate and guide, thus hindering the student’s learning experience due to a lack of dedicated teaching time. Moreover, not all clinicians are effective in or enjoy the role of clinical mentor. The free text responses showed that unconstructive criticism and outright hostility from the healthcare personnel was particularly prevalent in the data from Poland when compared to the rest of the five participating countries. That was identified as an important barrier to Polish students’ confidence in supporting physiological birth.

The socialization of a midwifery student into labor and birth care strongly influences their developing midwifery philosophy, values, beliefs and future midwifery identity^5^. Globally, the midwifery profession has a long-standing history of mentoring students and new graduate midwives^24^. Our findings suggest the relationship with the mentor midwife is critical to student confidence in supporting physiological birth in all study sites. Positive role modeling and a shared philosophy supported midwifery students’ confidence and personal power in supporting women-centered physiological birth^4^. However, if the mentors did not demonstrate belief in birth or practiced a more medicalized paradigm, the student–mentor relationship was a barrier to confidence in to supporting physiological birth. As mentors are often also the person assessing the midwifery students’ clinical performance, the mentors support may have impacted students’ attempts to build core midwifery skills that support physiology, such as therapeutic presence and shared decision-making. The way a midwife provides clinical care may be influenced by years of experience in a risk-focused and medicalized culture common at many hospital facilities, and may not be consistent with a physiological approach. In settings that are obstetric led and risk-focused, midwives’ attempts to support women targeting a physiological birth may be met with resistance and negative attitudes^25,26^. Medicalization of birth is reflected in the physical environment being constructed in a sterile, surgical-like environment rather than home-like in nature. The resultant physical and affective constraints on physiological birth were highlighted as a factor impacting student confidence in all five countries.

Many maternity settings are hierarchical with midwifery students perceiving that they hold a lower status. This perception can lead to disempowerment and stress, exacerbated by poor treatment of students by overworked midwives who are themselves feeling disempowered. Midwives able to function to the full extent of their education and expertise can enact physiological birth, even within medicalized work environments. However, this sometimes exacts an emotional toll on the midwife when the facility culture is hostile^27^. The experience of the Polish midwifery students possibly reflects this dynamic and its effect on student confidence. Mentor midwives unencumbered by disconfirming environments are more often able to support physiology when practicing in community birth settings. In this study, midwifery students identified exposure to low intervention, physiology-driven labor and birth as critical to building confidence to support normal birth. Some students noted that they were unable to achieve any real-life exposure or clinical participation in the experience of normal labor and birth whilst they were on their midwifery clinical placements.

The environment of birth is influenced by several factors that the midwifery students identified as influencing their confidence across settings. Staffing levels and the influence of other maternity care personnel were in the top four ranked factors mentioned with interprofessional support cited as key to physiological birth^28^. Hospitals whose maternity culture included routine risk averse medicalization, high obstetric intervention rates and fragmented maternity care systems were reported by students as decreasing their confidence in supporting physiological birth. This is reflected in the literature on midwives’ experiences of factors influencing physiological birth^29-31^. For the midwifery students in our study, the birth environment significantly impacted whether physiological birth was valued, supported and ultimately achieved across all five countries.

The top three factors revealed in this international study as being important to build midwifery student confidence in supporting normal birth are all factors found with the clinical placement. These factors relate to clinical personnel issues – the influence of mentors, staffing levels and other maternity care professionals. The fourth major factor is the theoretical education which informs the midwifery students’ practice, including their exposure to simulation practice sessions within the academic setting. Simulations enable midwifery students to practice and learn in an environment where errors can be made and corrected without serious consequence. This learning can be powerful and was mentioned by students as important in developing their confidence^32^.

Given the increase in medicalized intervention in the clinical setting, midwifery students may need to rely more than ever on the theoretical curricula, research evidence and clinical teaching/instruction to underpin their confidence surrounding physiological birth. The students affirmed the value of a strong evidence-based theoretical midwifery education that endorsed physiological birth. For some students with limited exposure to physiological birth in practice, the education component becomes their main source of knowledge and learning for this. The lack of coherence between the theoretical preparation students received at university and what they observed in practice was also highlighted, demonstrating the theorypractice gap dilemma and the conflicts students often felt when the midwifery care provided was not supported by quality evidence. This issue is commonly faced by experienced midwives as well^33^.

There were differences in some countries in factors influencing student confidence. For example, England and Australia are leaders in implementing midwifery continuity-of-care models to improve outcomes and experiences for childbearing families. The national drive to scale-up these models within these countries was reflected in the higher scores their students gave. Students in Northern Ireland and the USA ranked local labor and birth policies and laws higher than other countries. During the survey, local policies regarding induction were being debated in Northern Ireland, while in the US, state laws impact midwifery care by setting varying regulations for their scope of practice. Student confidence appears to be influenced by professional initiatives current during their midwifery education.

### Strengths and limitations

The variations in individual country sample sizes, and timing differences in data collection limit what can be concluded around the differences in country mean values for confidence levels amongst midwifery students. Logistical challenges of five countries with varying education pathways, healthcare systems, and practice restrictions are both limitations and strengths of this study. Understanding the country and context of the student midwife learning experiences with supporting physiological birth is critical to the interpretation of results. Even with these limitations, the consistency of findings around the factors which are most critical to building confidence is striking and can be useful in identifying supports for student midwives.

There was no psychometrically developed tool to measure midwifery student confidence for supporting normal birth, so the researchers created a survey. A Cronbach’s alpha of 0.69 was found for 10 variables in the survey representing factors that influence confidence, excluding the cohort variable. Within the survey, there are at least two constructs. Confidence in supporting normal birth is distinct from factors, such as policies or environment that influence confidence, and therefore the confidence variable was also excluded from the test.

The Likert scale scores ranged from 1 to 4; a larger numeric spread could provide finer discriminations in differences. Response bias is a possibility with a multiple item Likert scale. Confidence is behavior specific; supporting normal birth covers a variety of behaviors, such as providing therapeutic presence and comfort measures, for which an individual may have varying levels of confidence. Students were not provided with a definition of normal birth; this was left to their own understanding. Despite the limitations, this study provides robust data on midwifery student confidence to support normal birth that can be expanded and compared in future studies.

As convenience sampling was employed, sample selection bias is possible. Those who responded may be different than those who did not respond. As there was no tracking of individuals through their education, comparisons across phase of education are indicative only. We cannot draw conclusions from these data about the learning curves of individual midwifery students over time. A few midwifery students in England, Northern Ireland, and the USA responded to the survey at different stages of their education. As data were anonymized in the collection phase, it was not possible to identify which respondents may have participated more than once at the different data collection points, and therefore it was not possible to perform longitudinal analysis.

## CONCLUSIONS

Without adequate exposure to settings which promote physiology, midwives and students will struggle to develop the confidence to enact the core skills as outlined in the ICM International Definition of the Midwife and the ICM Essential Competencies for Midwifery Practice^34,35^. This is concerning not only for individual students’ confidence but also for the future of the midwifery profession, as historically a midwife’s area of expertise and authority of knowledge has been deeply rooted in physiological birth. The lack of midwifery skills to facilitate physiological birth will ultimately impact the childbearing experiences of those we serve. This perpetuates a vicious cycle of reduced opportunities for midwifery students to participate in physiological birth in their clinical setting. The relationship with a mentor midwife who is confident with the physiology of labor and birth, was reflected in the data as vital to student confidence. Pairing students with experienced, person-centered midwives who value exposing and educating midwifery students to normal birth can be instrumental in re-invigorating the hope of student midwives for the future of the profession and their place within it.

## Supplementary Material



## Data Availability

The data supporting this research are available from the authors on reasonable request.
